# Effects of sagging breasts and other risk factors associated with mastalgia: a case–control study

**DOI:** 10.1038/s41598-021-82099-2

**Published:** 2021-01-29

**Authors:** Bülent Çomçalı, Servet Kocaoz, Buket Altun Özdemir, Ömer Parlak, Birol Korukluoğlu

**Affiliations:** 1 Department of General Surgery, Breast Surgery Clinics, Ankara City Hospital, Bilkent, Üniversiteler Mah. Bilkent Cad., No: 1, Çankaya, 06800 Ankara, Turkey; 2 Department of General Surgery, Faculty of Medicine, Ankara Şehir Hastanesi, Ankara Yıldırım Beyazıt University, Bilkent, Üniversiteler Mah. Bilkent Cad., No: 1, Çankaya, 06800 Ankara, Turkey; 3 Department of General Surgery, Faculty of Medicine, Breast Surgery Clinics, Ankara Şehir Hastanesi, University of Health Sciences, Bilkent, Üniversiteler Mah. Bilkent Cad., No: 1, Çankaya, 06800 Ankara, Turkey

**Keywords:** Diseases, Medical research, Risk factors

## Abstract

The aim of this study is to compare patients with and without mastalgia and to analyze the factors affecting mastalgia and its severity. The patient’s age, height, weight, educational status, marital status, and occupation were recorded in all subjects. In addition, the women were asked about the presence of any risk factors for mastalgia, such as tea and coffee consumption, smoking, alcohol consumption, and weight gain. The sternal notch to nipple distance (SNND) was measured to determine whether there was breast sagging. Mastalgia was significantly more common in women with BMIs of > 30 kg/m^2^ (OR: 2.94, CI 1.65–5.24), those who were primary school graduates or illiterate (OR: 2.96, CI 1.6–5.46), and those with SNND values of 22–25 cm (OR: 2.94, CI 1.79–4.82). In these women, drinking more than 6 cups of tea a day (OR: 2.15, CI 1.32–3.5), smoking at least 10 cigarettes a day (OR: 2.94, CI 1.78–4.83), and drinking alcohol at least once a week (OR: 2.1, CI 1.12–3.91) were found to be important factors that increased the risk of mastalgia. As a result, it has been found that severe mastalgia complaints cause by obesity, sagging breasts, never giving birth, unemployment anxiety, regular smoking, alcohol use, and excessive tea consumption.

## Introduction

Mastalgia is a pain felt in the breast tissue. Women frequently apply to primary and secondary health care institutions due to this complaint. In most women, the pain lasts a few days to a month and disappears on its own. When patients with mastalgia learn that they do not have cancer via examination and imaging methods, the vast majority feel relieved and only 15% of them require medical treatment^1,2^. Mastalgia usually occurs in women between the ages of 30 and 50. Cyclic or periodic mastalgia is related to the menstrual cycle and is common in young women. It is felt before menstruation usually in the upper outer quadrant of both breasts. This pain is common and can spread to the arms and armpits. It usually disappears with the onset of menstruation^3,4^. Non-cyclic mastalgia is independent of the menstrual cycle. It is most commonly seen between the ages of 40 and 50 and is felt in the form of a sharp burn. Non-cyclic mastalgia is also sometimes limited, mostly unilateral, and felt only in a quadrant of the breast^5,6^. Cancer-related mastalgia is rare. It is seen as unilateral and well localized, not associated with the menstrual cycle^7,8^. Along with smoking, excessive caffeine intake, obesity, alcohol consumption, pregnancy, mastitis, trauma, canal ectasia, macrocysts of the breast, and benign tumors can also cause mastalgia^8–10^. Additionally, gaining weight in the last 5 years is one of the common factors that can cause mastalgia^10^. In previous studies, the effects of breast size and the bra used on mastalgia were investigated^6–10^. However, there is no study in the literature investigating the effect of breast sagging on mastalgia. Therefore, the aim of our study is to compare patients with and without mastalgia to investigate the factors affecting mastalgia and the effects of breast sagging, which we hypothesize is one of these factors.

## Materials and methods

A total of 440 women including 220 with mastalgia and 220 without who applied to Ankara City Hospital’s breast and endocrine surgery outpatient clinic with breast disorders or other complaints (thyroid or parathyroid) participated in this study. Women who agreed to participate in the study were administered a questionnaire for mastalgia assessment after completing the informed consent form. The study was carried out between January 1, 2020, and March 30, 2020. In addition to demographic and socioeconomic questions, in this study, a total of 26 questions were asked, along with 11 questions regarding possible risk factors. The questionnaire was applied by the physicians who conducted the study. With the questionnaire on demographic and socioeconomic data, the patient’s age, height, weight, educational background, marital status, and profession were recorded. The sternal notch to nipple distance (SNND) was measured while the patient was sitting in an upright position. In addition, women were asked if they had babies and about the breastfeeding period. They were also asked about the presence of mastalgia, its severity, and its relationship with menstruation. While 63.6% (n = 140) of the patients with mastalgia had periodic pain, 36.4% (n = 80) reported non-periodic pain. In the questionnaire applied to the patients, a 10-cm visual pain scale is used to measure pain, from “no pain” on one end to “the most severe pain possible” on the other. The length of the distance from the place where “no pain” is marked on this scale to the point marked by the patient indicates the patient’s level of pain^11^. Patients experiencing mastalgia were asked to score the severity of this pain from 1 to 10 as a visual analogue scale (VAS) pain score^12^. In addition, it was asked whether there were any risk factors related to mastalgia such as tea and coffee consumption, smoking, alcohol consumption, and weight gain.

The following patients were not included in our study: patients who were not between 18 and 65 years of age, those who had psychiatric disorders such as depression and anxiety disorder, those who had experienced physical or psychological trauma (such as a traffic accident or loss of relatives), those who refused to volunteer to work with patients with chronic illness, and those with neurological disorders and mental or physical disabilities.

In this case–control study, the sample size was determined using G-power software version 3.1.9.4. According to the G-power analysis, it was planned to use the chi-square test to compare the risk factors that cause mastalgia in women. This test was used to calculate the medium effect size (d = 0.5) and 95% power level. It was found necessary to enroll a total of at least 220 patients in the study. As a result, it was projected that 440 women, 220 with mastalgia and 220 without mastalgia, would be included in the study. This case–control study is hospital-based.

### Statistical analysis

The data obtained from the study were uploaded to the SPSS program via computer and analyzed (version 25.0; IBM Corp., Armonk, NY, USA). The body mass index (BMI) for each woman was calculated by dividing the weight by the square of the height (kg/m^2^). Number, percentage, mean, median, interquartile range, and standard deviation values were used to evaluate descriptive statistics. The Kolmogorov–Smirnov test was used to check whether age, BMI, and SNND data, which were non-categorical parameters of the case and control groups, were normally distributed. It was understood that the data did not show normal distribution since the test results were p < 0.05. The Mann–Whitney U test was used to compare these groups. Pearson chi-square tests were used for the analysis of categorical variables. Post-hoc comparisons were used to analyze where differences appeared in the comparison of subgroups. Risk factors for mastalgia were evaluated by binary logistic regression analysis. Receiver operating characteristic (ROC) curve testing was used to determine the appropriate positive threshold of the categorical variables. For the retrospective model, the inclusion criterion was accepted as 0.01 and the exclusion criterion as 0.05. Independent variables were included in the analysis by coding.

While performing logistic regression analysis, previous studies were taken as examples^10,13^. ROC curve testing was used to determine the age variable’s appropriate positive threshold. According to the ROC analysis, mastalgia was significantly more common in patients 36 years and older. Unlike in previous studies, SNND measurement was performed in our work. The SNND values in women without breast sagging are between 19 and 21 cm^13,14^, and mastalgia was significantly more common in women in the group with SNND values of 22–25 cm (Pearson chi-square test value of 31.07, p < 0.001). Regression model data fit and multiple linearity problems were evaluated. The validity of the models obtained was tested with the Hosmer–Lemeshow test.

Before conducting regression analysis, the criteria were coded as follows: age: 36–45 years (1), other age groups (0); BMI: > 30 (1), others (0); SNND value: 22–25 (1), others (0); Educational status: Illiterate and primary education graduate (1), others (0); Working status: Unemployed woman (1), employed (0); Weight gain status: those gaining weight in the past 5 years (1), those not gaining (0); First menstrual age: first menstrual age < 12 (1), others (0); giving birth: two births or more (1), others (0); age of first birth: first birth experience at the age of 30 or older (1), others (0); breastfeeding status: breastfeeding (1), not breastfeeding (0); tea consumption: drinking 6 cups of tea per day (1), others (0); smoking: more than 10 cigarettes per day (1), others (0); alcohol use: drinking at least 1 day per week (1), others (0).

### Ethical approval

The study was found ethically appropriate by the decision of the Ankara City Hospital Ethics Committee, dated 16.01.2019 and numbered 259. After the approval of the ethics committee was received, a questionnaire was applied to the patients who applied to our outpatient clinic and agreed to participate. Before the survey, women were briefly informed about the study. The data of the study were obtained from the questionnaires. The research was carried out in line with the Declaration of Helsinki.

## Results

A total of 440 patients were examined. The mean age of the patients was recorded as 36.18 ± 11.03 among those without mastalgia, while it was 37.60 ± 11.53 in patients with mastalgia complaints (Table 1). The mean BMI value of patients without mastalgia was recorded as 25.92 ± 5.83 kg/m^2^, while in patients with mastalgia it was 29.71 ± 6.96 kg/m^2^ (p < 0.001). The mean SNND value in patients without mastalgia was 22.11 ± 2.77 cm and the mean SNND value in patients with mastalgia was 23.73 ± 3.59 cm. Thus, the SNND value was significantly higher in patients with mastalgia (p < 0.001). Periodic mastalgia associated with the menstrual cycle was significantly more frequent and was found in 63.6% (n = 140) of the patients with mastalgia (p < 0.001). Mastalgia was observed more frequently in the lower outer and upper outer quadrants of the breasts (Table 2). It was observed that patients with mastalgia had been suffering from this complaint for a mean of 9.92 ± 21.51 months. Pain scores (VAS scores) of patients with mastalgia complaints were found to be 4.92 ± 2.01. It was determined that women who were unemployed, which is one of the risk factors of mastalgia, had a higher rate of mastalgia compared to those employed (p < 0.001) (Table 3). University graduates were found to have less mastalgia than those with other educational statuses (p < 0.001). Mastalgia was found to be significantly more common in married women than in single women (p = 0.017). It was also statistically significant that mastalgia was more common in women who were breastfeeding (p = 0.004). Likewise, mastalgia was significantly more common in women who had given birth to 2 or more babies (p < 0.001). There was a significant relationship between weight gain in the last 5 years and mastalgia (p < 0.001). Mastalgia was more common in women who consumed alcohol than in those who did not (p = 0.001). Mastalgia was also more common in smokers than non-smokers (p < 0.001) (Table 4), as well as in those who consumed tea excessively (p < 0.001).

**Table 1:** Comparison of body mass index measurements of those with and without mastalgia.

	Not having mastalgia				Having mastalgia				Test/p
	Median	Min	Max	IQR	Median	Min	Max	IQR	
Age	35.50	19	61	19	37.50	19	61	18	0.213
BMI	25.39	15.23	53.33	7.85	28.67	18.34	51.56	9.5	< 0.001
SNND	21	18	30	4	23	18	34	4	< 0.001

Mann–Whitney U test was used.

*Min.* minimum, *Max.* maximum, *SD* standard deviation, *IQR* interquartile range, *BMI* body mass index, *SNND* distance from the sternal notch to the nipple.

**Table 2:** Localization of the pain in the breasts of women with mastalgia.

	n	%
**Location of the mastalgia**		
Both breasts	116	52.7
Right breast only	80	36.4
Left breast only	24	10.9
**Breast quadrant where the mastalgia is felt**		
Lower inner quadrant	26	11.8
Lower outer quadrant	50	22.7
Upper inner quadrant	8	3.6
Upper outer quadrant	54	24.5
On the nipple	30	13.6
Generally on the breast	52	23.6
Total	220	100

*n* number.

**Table 3:** Distribution of certain variables in groups with and without mastalgia.

Risk factors	Not having mastalgia		Having mastalgia		Test/p
	n	%	n	%	
**Educational background**					
≤ Primary school graduate	18	22.5	62	77.5	< 0.001
Secondary school graduate	14	33.3	28	66.7	
High school graduate	46	47.9	50	52.1	
≥ University graduate	142	64.0	80	36.0	
**Employment**					
Unemployed	62	35.6	112	64.4	0.001
Employed	158	59.4	108	40.6	
**Marital status**					
Single	90	57.7	66	42.3	0.017
Married	130	45.8	154	54.2	
**Number of births**					
Never had a baby	106	58.2	76	41.8	< 0.001
Gave birth to 1 baby	36	60.0	24	40.0	
Gave birth to 2 babies or more babies	78	39.4	120	60.6	
**Breastfeeding status**					
Breastfeeding	108	43.9	138	56.1	0.004
Not breastfeeding	112	57.7	82	42.3	
**Smoking**					
More than 10 cigarettes a day	68	37.8	112	62.2	< 0.001
Nonsmoker	152	58.5	108	41.5	
**Alcohol**					
Drinking alcoholic beverages at least 1 day a week	28	33.3	56	66.7	0.001
Nondrinker	192	53.9	164	46.1	
**Consumption of tea on a daily basis**					
0–2 cups	72	70.6	30	29.4	< 0.001
3–5 cups	84	56.8	64	43.2	
6–8 cups	46	37.1	78	62.9	
9 or more	18	27.3	48	72.7	
**Gaining weight**					
Gaining	124	43.4	162	56.6	< 0.001
Not gaining	96	62.3	58	37.7	

Pearson chi-square test was used.

*n* number.

**Table 4:** Logistic regression model of risk factors—enter method results.

	Exp (B) Odds ratio	95% CI for Exp (B)	
		Lower	Upper
Age 36–45	2.81	1.54	5.13
BMI > 30	2.94	1.65	5.24
SNND 22–25 cm	2.94	1.79	4.82
Primary school graduate or illiterate	2.96	1.60	5.46
Smoking at least 10 cigarettes daily	2.94	1.78	4.83
Drinking 6 or more cups of tea daily	2.15	1.32	3.50
Drinking alcohol at least once weekly	2.10	1.12	3.91
Unemployed	1.74	1.03	2.93
Gaining weight in the last 5 years	1.38	0.84	2.28
Breastfeeding	1.02	0.55	1.89
Never had a baby	1.85	0.97	3.52
First menstrual period age younger than 12	1.34	0.85	2.12

Pseudo (Nagelkerke) R^2^ = 0.368, Hosmer–Lemeshow χ^2^ = 26.121, p = 0.001.

*CI* confidence interval, *OR* odds ratio, *BMI* body mass index, *SNND* distance from the sternal notch to the nipple.

As a result of the post-hoc comparison for educational status, the difference between the subgroups was found to be due to the difference between university graduates and the others, and between the primary school graduate or illiterate group and the others. As a result of the post-hoc comparison for number of cups of tea consumed per day, there was no difference between drinking 6–8 cups of tea per day and drinking 9 or more cups of tea. It was observed that there was a significant difference between these groups and the other groups. Finally, a post-hoc comparison revealed a significant difference between those who gave birth at all and those who had 2 or more births.

The risk of having periodic mastalgia complaints was increased among women with BMIs of > 30 kg/m^2^ by 1.7 times, among those smoking at least 10 cigarettes a day by 2.9 times, among those drinking more than 6 cups of tea daily by 2.1 times, and among those who never had a baby by 2.1 times (Table 5). The risk of having non-periodic mastalgia complaints was increased among women with SNND values of 22–25 cm by 3.4 times, among primary school graduates or illiterate women by 4 times, among those consuming alcohol at least once a week by 3.7 times, among those worrying about being unemployed by 2.5 times, and among those who never had a baby by 2.1 times (Table 6). The risk of having severe mastalgia complaints was increased among women with BMIs of > 30 kg/m^2^ by 5.9 times, among those with SNND values of 22–25 cm by 2.4 times, among those smoking at least 10 cigarettes a day by 4.1 times, among those consuming alcohol at least once a week by 54 times, among those worrying about being unemployed by 2.6 times, and among those who never had a baby by 3.5 times (Table 7).

**Table 5:** Logistic regression model of risk factors (periodic mastalgia)—backward method results.

	Exp (B) Odds ratio	95% CI for Exp (B)	
		Lower	Upper
BMI > 30	1.72	1.02	2.93
Age 36–45	1.62	0.96	2.71
First menstrual period age younger than 12	1.53	0.99	2.37
Smoking at least 10 cigarettes daily	2.86	1.82	4.49
Drinking 6 or more cups of tea daily	2.14	1.34	3.40
Never had a baby	0.51	0.30	0.86

Pseudo (Nagelkerke) R^2^ = 0.164, Hosmer–Lemeshow χ^2^ = 40.723, p < 0.001 (Step 7).

Dependent variable: 1: mastalgia absent; 0: mastalgia present.

*CI* confidence interval, *OR* odds ratio, *BMI* body mass index.

**Table 6:** Logistic regression model of risk factors (non-periodic mastalgia)—backward method results.

	Exp (B) Odds ratio	95% CI for Exp (B)	
		Lower	Upper
SNND 22–25 cm	3.44	1.80	6.57
Primary school graduate or illiterate	3.96	1.86	8.45
Drinking alcohol at least once weekly	3.71	1.67	8.25
Unemployed	2.48	1.20	5.15
Gaining weight in the last 5 years	3.49	1.65	7.38
Never had a baby	10.27	4.38	24.08

Pseudo (Nagelkerke) R^2^ = 0.465, Hosmer–Lemeshow χ^2^ = 21.484, p = 0.006 (Step 7).

Dependent variable: 1: mastalgia absent; 0: mastalgia present.

*CI* confidence interval, *OR* odds ratio, *SNND* distance from the sternal notch to the nipple.

**Table 7:** Logistic regression model of risk factors (severe mastalgia)—backward method results.

	Exp (B) Odds ratio	95% CI for Exp (B)	
		Lower	Upper
BMI > 30	5.87	2.08	16.54
SNND 22–25 cm	2.41	1.08	5.36
Smoking at least 10 cigarettes daily	4.14	1.69	10.12
Drinking 6 or more cups of tea daily	0.47	0.20	1.15
Drinking alcohol at least once weekly	54.07	18.70	156.31
Unemployed	2.57	1.14	5.83
Never had a baby	3.53	1.33	9.37
First menstrual period age younger than 12	0.47	0.21	1.07

Pseudo (Nagelkerke) R^2^ = 0.541, Hosmer–Lemeshow χ^2^ = 38.397, p < 0.001. (Step 6).

*CI* confidence interval, *OR* odds ratio, *BMI* body mass index, *SNND* distance from the sternal notch to the nipple, *Severe mastalgia* VAS score ≥ 8.

BMI of > 30 kg/m^2^, smoking more than 10 cigarettes per day, drinking more than 6 cups of tea daily, and never having had a baby explained 16.4% of the causes of periodic mastalgia (p < 0.001). SNND values of 22–25 cm, being a primary school graduate or illiterate, consuming alcohol at least once a week, worrying about being unemployed, and never having had a baby explained 46.5% of the causes of non-periodic mastalgia (p = 0.006). BMI of > 30 kg/m^2^, SNND values of 22–25 cm, smoking at least 10 cigarettes a day, consuming alcohol at least once a week, worrying about being unemployed, and never having had a baby explained 54.1% of the causes of severe mastalgia (p < 0.001).

## Discussion

In our study, the issue of age range was approached carefully, ensuring that there was no significant difference between the groups in terms of average age in order to analyze the risk factors of patients with and without mastalgia. In a previous community-based study, mastalgia complaints were reported to be more common in women aged 35–50^10^.

We found that patients with BMI levels of 30 and above experienced mastalgia 1.7 times more frequently than other women. In previous studies, similar to our results, it has been reported that mastalgia complaints were 2 times more common in obese women^10,13^. Obesity is a risk factor for mastalgia and losing weight has a positive effect on it^13^. In our study, we found that there were mild or no mastalgia complaints among women who had lost weight and returned to their ideal weight, but, on the other hand, they experienced excessive breast sagging.

Large, sagging breasts can cause pain due to the stretching of Cooper’s ligaments. It has been reported that mastalgia complaints are frequently seen in women with macromastia and that a sports bra supporting the breasts results in less suspension, thus alleviating mastalgia complaints^15,16^. Similarly, in our study, women with sagging breasts with SNND values of 22–25 were found to experience non-periodic mastalgia 3.4 times more frequently.

The impact of excessive tea consumption and caffeine intake on mastalgia is controversial. Women who consume tea and coffee excessively have a 4–5 times higher risk of mastalgia than other women, and it has been reported that reducing the amount of caffeine in the diet is useful in the treatment of mastalgia^17^. Stress, caffeine, smoking, frequency of breastfeeding, and benign breast diseases are all associated with mastalgia in previous studies^9^. In our study, it was found that the complaint of mastalgia was twice as high among women who consumed 6 cups of tea or more a day. Also, in women who smoked at least 10 cigarettes per day and those who consumed alcohol once a week, mastalgia complaints were seen an average of 3 times more often than in other women. Johnson et al. reported that mastalgia complaints were observed in women drinking alcohol twice as often^13^. In some previous studies, it was reported that drinking tea and smoking had no effect on mastalgia, but reducing caffeine intake would be beneficial for it^10,18^. Methyl-xanthine is found in products such as coffee, chocolate, black tea, and cola drinks, and there is a significant decrease in the sensitivity of breast tissue in more than 97% of women who completely avoid methyl-xanthine^19^.

In our study, the complaint of mastalgia was 4 times more frequent in illiterate women or primary school graduates than others. However, in women who did not work, although the rate of mastalgia complaints was high, no significant results were found in regression analysis. One study reported that mastalgia complaints were higher among high school graduates^10^, while another study reported that mastalgia was more common in illiterate women or primary school graduates^18^. In our study, mastalgia was more common among primary school graduates.

In one study, it was reported that mastalgia complaints were more frequent in married women^13^. In our study, there were statistically significant differences for the marital status of women, giving birth to 2 or more babies, breastfeeding, and gaining weight in the last 5 years and mastalgia. However, there were no significant results in regression analysis. In another study, it was reported that mastalgia complaints were significantly more frequent in women who breastfed 3 or more babies^9^. In addition, another study reported that mastalgia complaints were significantly more frequent in women who gained weight over the past 5 years and that breastfeeding was not associated with mastalgia^10^.

The strength of this study is that it was conducted by the physicians of a breast and endocrine surgery clinic. In addition, it is the assessment of mastalgia severity using physical examination and the VAS scale. It is a careful examination of various risk factors, including “sternal notch to nipple distance”. The limitations of the study are that lifestyle, eating habits, physical activity, stress level, and drug treatments are not included in the study and that the study did not include patients with psychiatric disorders. The study is a hospital-based study rather than a community-based study.

In conclusion, it was found that mastalgia was significantly more common in women who were obese, as well as those with sagging breasts, smokers, those drinking alcohol, and those consuming excessive amounts of tea. Mastalgia was found to be more periodic (related to the menstrual cycle) in these patients. It has been determined that mastalgia is more common in the lower outer quadrant of the breast and in the upper outer quadrant of the breast. Women who are illiterate or who are primary school graduates complain more about mastalgia. It has been found that severe mastalgia complaints cause by obesity, sagging breasts, never giving birth, unemployment anxiety, regular smoking, alcohol use, and excessive tea consumption.
